# HIV-1 subtype diversity, transmission networks and transmitted drug resistance amongst acute and early infected MSM populations from Coastal Kenya

**DOI:** 10.1371/journal.pone.0206177

**Published:** 2018-12-18

**Authors:** Amin S. Hassan, Joakim Esbjörnsson, Elizabeth Wahome, Alexander Thiong’o, George N. Makau, Mathew A. Price, Eduard J. Sanders

**Affiliations:** 1 KEMRI/Wellcome Trust Research Programme, Kilifi, Kenya; 2 Lund University, Lund, Sweden; 3 Oxford University, Oxford, United Kingdom; 4 International AIDS Vaccine Initiative, New York, New York, United States of America; 5 Department of Epidemiology and Biostatistics, University of California at San Francisco, San Francisco, California, United States of America; Fudan University, CHINA

## Abstract

**Background:**

HIV-1 molecular epidemiology amongst men who have sex with men (MSM) in sub-Saharan Africa remains not well characterized. We aimed to determine HIV-1 subtype distribution, transmission clusters and transmitted drug resistance (TDR) in acute and early infected MSM from Coastal Kenya.

**Methods:**

Analysis of HIV-1 partial *pol* sequences from MSM recruited 2005–2017 and sampled within six months of the estimated date of infection. Volunteers were classified as men who have sex with men exclusively (MSME) or with both men and women (MSMW). HIV-1 subtype and transmission clusters were determined by maximum-likelihood phylogenetics. TDR mutations were determined using the Stanford HIV drug resistance database.

**Results:**

Of the 97 volunteers, majority (69%) were MSMW; 74%, 16%, 9% and 1% had HIV-1 subtypes A1, D, C or G, respectively. Overall, 65% formed transmission clusters, with substantial mixing between MSME and MSMW. Majority of volunteer sequences were either not linked to any reference sequence (56%) or clustered exclusively with sequences of Kenyan origin (19%). Eight (8% [95% CI: 4–16]) had at least one TDR mutation against nucleoside (n = 2 [2%]) and/or non-nucleoside (n = 7 [7%]) reverse transcriptase inhibitors. The most prevalent TDR mutation was K103N (n = 5), with sequences forming transmission clusters of two and three taxa each. There were no significant differences in HIV-1 subtype distribution and TDR between MSME and MSMW.

**Conclusions:**

This HIV-1 MSM epidemic was predominantly sub-subtype A1, of Kenyan origin, with many transmission clusters and having intermediate level of TDR. Targeted HIV-1 prevention, early identification and care interventions are warranted to break the transmission cycle amongst MSM from Coastal Kenya.

## Background

Kenya’s over three decades long HIV-1 epidemic is characterized by both heterosexual and homosexual transmission [1, 2]. Compared to the general male population, men who have sex with men (MSM) are disproportionately affected, with an HIV-1 prevalence that is two- to four-fold higher [3]. Whilst debatable, mathematical modelling has suggested that MSM may substantially contribute to the HIV-1 epidemic in the general population in Kenya [4, 5]. Phylogenetic methods offer the potential to disentangle HIV-1 transmission dynamics and contribution of MSM to the general population at a molecular level and inform prevention efforts [6–8]. Only one study has assessed phylogenetic linkages between MSM and the heterosexual population in Kenya [9]. This study reported sexual interaction between and within MSM from Nairobi and Coastal Kenya, but minimum mixing between MSM and the general male and female population between and within both locations. These, and findings from a study done in Senegal [10, 11], the only other study that has reported the molecular characteristics of the HIV-1 MSM epidemic in sub-Saharan Africa (sSA), suggest the existence of a HIV-1 MSM epidemic that may be separate and different from that in the general heterosexual population.

MSM have heterogeneous HIV-1 acquisition risks. In a study from coastal Kenya, men who have sex with men exclusively (MSME) had a six-fold higher HIV-1 incidence compared to men who have sex with both men and women (MSMW) [12]. The higher HIV-1 acquisition risk amongst MSME, compared to MSMW, may be attributed to either behavioral or viral molecular differences. Yet, molecular characterization of the HIV-1 MSM epidemic, and specifically transmission networks between MSME and MSMW, remains less well-studied in sub-Saharan Africa (sSA).

In Kenya, several studies have described HIV-1 subtype distribution in the general heterosexual population, and reported a predominance of HIV-1 sub-subtype A1 [13–18], with increasing inter-subtype recombinants [19–22]. In addition, a study amongst the heterosexual population from Coastal Kenya reported minimum clustering and high genetic variability between the study-volunteers’ *pol* sequences [21]. Given that HIV-1 MSM epidemics may be separate and different from the general population epidemic, and different acquisition risks exists between MSME and MSMW, it is uncertain if HIV-1 MSM sequences, and specifically those between MSME and MSMW, share the same subtype distribution and clustering properties as that reported from the general heterosexual population.

HIV-1 transmitted drug resistance (TDR) amongst the general HIV-1 infected population has also been extensively characterized in Kenya, with reports of low (<5%) to moderate (5–15%) TDR levels [23–28]. Generally, studies involving acute or early infected individuals tend to report higher TDR levels compared to those from chronically infected participants, even when estimated from the same geographic setting [23, 24, 27]. A temporal increase in TDR estimates amongst the general population has been documented, with one study reporting an increase from 3.9% in 2006 to 10.9% in 2014 in Kenya [26].

Importantly, there are hardly any data that describe TDR amongst MSM in Kenya. Only one multinational observational study that included MSM data and samples collected between 2006 and 2009 exists and reported a TDR prevalence of 4% (2/49) from Coastal Kenya [28]. In the current study, we expand work from this study and provide an update on TDR estimates. We also aimed to characterize HIV-1 subtype diversity and transmission networks among this acute and early infected MSM cohort, further characterized into MSME and MSMW, from Coastal Kenya.

## Methods

### Study design

Data and samples were obtained from a prospective observational study following high-risk volunteers in a HIV-1 vaccine feasibility study in Coastal Kenya. MSM volunteers, further characterized into MSMW and MSME, were recruited between 2005 and 2017, and followed monthly or quarterly as previously described [12].

Identification of acute and early infection in this cohort has also been described in detail elsewhere [12]. In brief, HIV-1 testing was performed at each study visit using two rapid antibody test kits in parallel (Determine, Abbott Laboratories; Unigold, Trinity Biotech). Discordant results were resolved using an enzyme-linked immunosorbent assay (ELISA, Genetic System HIV-1/2 plus O EIA; Bio-Rad). All HIV-1 negative or discordant samples were tested for p24 antigen (Vironostika HIV-1 p24 ELISA; Biomerieux), and pre-seroconversion and post-seroconversion samples were tested for HIV-1 ribonucleic acid (RNA) (Amplicor Monitor 1.5; Roche).

HIV-1 antibody test results were relayed to volunteers in real time. All volunteers testing HIV-1 negative were supported with risk reduction counselling. Those testing HIV-1 positive were either enrolled for follow up care in other early infection studies [29, 30], or referred to their proximate clinics of choice for follow up care and antiretroviral therapy (ART).

In Kenya, ART was rolled out in public health facilities in 2006, with eligibility based on a pre-defined CD4+ T-cell count or WHO clinical staging criteria. Standard first-line regimen included two nucleoside reverse transcriptase inhibitors (NRTI) and a non-nucleoside reverse transcriptase inhibitor (NNRTI). Individuals failing first-line regimen were switched to a second line regimen comprising two NRTIs and a protease inhibitor (PI) [31]. Immediate ART initiation, regardless of the CD4 T-cell count or WHO clinical staging, was recommended from 2016 [32].

For the purpose of our analysis, the earliest HIV-1 infected samples (or HIV-1 *pol* sequence, where available) from volunteers diagnosed with acute and early infection, defined as samples collected within 6 months of the estimated date of infection (EDI), were considered [33]. Overall, 97 MSM volunteers met our eligibility criteria and were included in the analyses.

### HIV-1 genotyping

Of those included in our analyses, samples from 81 volunteers had available HIV-1 *pol* sequence data (genotyping details published elsewhere) [28]. Genotyping for the remaining 16 samples were done as follows: HIV-1 RNA was extracted from 100 μl of blood plasma using the RNeasy lipid tissue mini kit (Qiagen). HIV-1 RNA were reverse transcribed and PCR amplified using the one-step Superscript III RT/Platinum Taq High Fidelity protocol (Invitrogen) according to manufacturer’s instructions, with *pol* primers JA269-JA272 [34]. A nested PCR with primers JA270 and JA271 was then done using Dream Taq DNA polymerase (5/UL) (ThermoFisher Scientific) according to manufacturer’s instructions. Successfully amplified PCR products were confirmed by agarose gel electrophoresis and prepared for sequencing using the inner primers (JA270 and JA271) and the Big Dye terminator kit (Applied Biosystems). These were processed using the 3130 genetic analyzers (Applied Biosystems).

### HIV-1 subtype determination

The forward and reverse fragments were assembled using Sequencher (v5.4.6) and saved in a consensus FASTA file. All sequence files were aligned using the Clustal algorithm in MEGA7 [35]. In addition, the most recent (2010) HIV-1 subtype reference sequence dataset was obtained from the Los Alamos HIV sequence database (https://www.hiv.lanl.gov/content/index). A profile alignment was done in Clustal X2 (v 2.1) [36] for the volunteer and the reference sequences. The combined volunteer and reference sequence alignment was edited in MEGA7 and submitted for phylogenetic reconstruction using the general time reversible (GTR) model of nucleotide substitution with gamma distributed rate heterogeneity. Branch support was assessed using the Shimodaira-Hasegawa like approximate Likelihood Ratio Test (aLRT-SH) on the PhyML online portal [37]. The resulting phylogenetic tree was viewed in Figtree (v1.4.3) (http://tree.bio.ed.ac.uk/software/figtree/), with branch support of aLRT-SH ≥ 0.90 considered significant [38].

### Transmission clusters

Based on the subtyping results above, sequences were grouped into the main subtypes observed. For each subtype-specific dataset, a search for related sequences was done separately using the NCBI GenBank BLAST tool [39], with results limited to a threshold of 10 similar hits per volunteer sequence. Duplicate sequences were removed based on the sequence identifiers and accession numbers. Redundant sequences were then removed using Skipredundant on EMBOSS (http://www.bioinformatics.nl/cgi-bin/emboss/skipredundant). Every single hit was further explored to identify and exclude previously published volunteer sequences.

Overall, 330 reference sequences were identified (S1 Table). These were aligned in turn with volunteer subtypes A, C and D sequences, and submitted for phylogenetic analysis as outlined above. Clusters were identified using Cluster Picker [40]. Branch support of aLRT-SH ≥0.90 and a genetic distance of ≤0.06 were considered acceptable to infer transmission clusters [41]. Active transmission clusters were further explored using aLRT-SH branch support of ≥ 0.900 and genetic distance ≤0.015 [41]. Transmission networks were defined based on the number of MSM sequences as dyads (2 sequences) and networks (≥ 3 sequences) [38, 42].

### Transmitted drug resistance

Volunteer sequences were submitted to the Stanford HIV drug resistance database using the calibrated population resistance tool to screen for *pol* resistance-associated mutations (http://cpr.stanford.edu/cpr.cgi). Transmitted resistance mutations were identified based on the WHO list for surveillance of genotypic drug resistance mutations [43]. The prevalence of transmitted resistance was estimated, and their 95% binomial confidence intervals (CI) presented. In addition, phylogenetic analysis of volunteer and reference sequences was repeated, as described above, to assess for clustering amongst isolates identified with surveillance drug resistance mutations.

### Data analysis

Continuous data were presented using medians and interquartile ranges (IQR). Age was further stratified into two categories based on the median value as youth/younger adults (<24.9 years) and older (≥25.0 years) participants. Categorical data were presented using frequencies and percentages. Associations in continuous data were determined using the non-parametric rank-sum test. Associations in categorical data were determined using the Pearson’s chi-squared test. All analyses were done using Stata I/C 15.0 (StataCorp LLC).

### Ethical considerations

The study received ethics approval from the Kenya Medical Research Institute (KEMRI) Scientific and Ethics Review Unit (parent protocol numbers. SSC 894 and SSC 1027). All volunteers provided written informed consent. All consensus HIV-1 *pol* sequence fasta files are available from GenBank (Accession numbers MK192535—MK192631).

## Results

### Characteristics of study participants

Of the 97 HIV-1 infected MSM volunteers, majority (n = 67 [69%]) were MSMW. MSME volunteers were younger compared to MSMW volunteers (22.3 [IQR: 21.1–25.5] vs. 24.5 [IQR: 22.2–28.2] years respectively, p = 0.010). Overall, a majority of the volunteers had a secondary/higher level of education, had their samples collected in 2009/12, had not recently engaged in group sex, had a history of recent transactional sex, reported recent alcohol use and had ≤ 2 recent sex partners. There were no significant differences in the distribution of MSME and MSMW by education status (p = 0.359), year of sample collection (p = 0.480), group sex (p = 0.628), transactional sex (p = 0.345), alcohol use (p = 0.248) and total sex partners (0.606) (Table 1).

**Table 1:** Demographic and risk behavior distribution of men who report having sex with men (MSM) volunteers with acute and early HIV-1 infection from Coastal Kenya, 2005–2017 (N = 97).

Characteristics	Categories	MSME, n = 30 (%)	MSMW, n = 67 (%)	MSM, n = 97 (%)
Age at EDI (years, median [IQR])		22.3 [21.1–25.5]	24.5 [22.2–28.2]	24.0 [21.7–27.1]
Age group at EDI (years)	18.0–24.9 25.0–39.9	21 (70.0) 9 (30.0)	36 (53.7) 31 (46.3)	57 (58.8) 40 (41.2)
Education status	Primary, lower Secondary, higher	16 (53.3) 14 (46.7)	29 (43.3) 38 (56.7)	45 (46.4) 52 (53.6)
Year of sample collection	2005/08 2009/12 2013/17	8 (26.7) 13 (43.3) 9 (30.0)	18 (26.9) 36 (53.7) 13 (19.4)	26 (26.8) 49 (50.5) 22 (22.7)
EDI to sample collection (months)		0.8 (0.5–1.4)	0.8 (0.5–1.5)	0.8 (0.5–1.5)
EDI to sample collection categories	Less than 1 month More than 1 month	17 (56.7) 13 (43.3)	34 (50.7) 33 (49.3)	51 (52.6) 46 (47.4)
*Group sex	No Yes	25 (83.3) 5 (16.7)	53 (79.1) 14 (20.9)	78 (80.4) 19 (19.6)
*Transactional sex	No Yes	7 (23.3) 23 (76.7)	22 (32.8) 45 (67.2)	29 (29.9) 68 (70.1)
**Alcohol use	No Yes	14 (46.7) 16 (53.3)	23 (34.3) 44 (65.7)	37 (38.1) 60 (61.9)
***Total sex partners	≤ 2 partners > 2 partners	23 (76.7) 7 (23.3)	48 (71.6) 19 (28.4)	71 (73.2) 26 (26.8)

Abbreviations: EDI (estimated date of infection), IQR (inter-quartile ranges), MSME (men who have sex with men, exclusively) and MSMW (men who have sex with men, and women).

*Group sex/transactional sex in the three months preceding the positive HIV-1 diagnosis.

**Alcohol use in the one month preceding the positive HIV-1 diagnosis

***Total sex partners: in the one week preceding the positive HIV-1 diagnosis

### HIV-1 subtype determination

**Fig 1:**
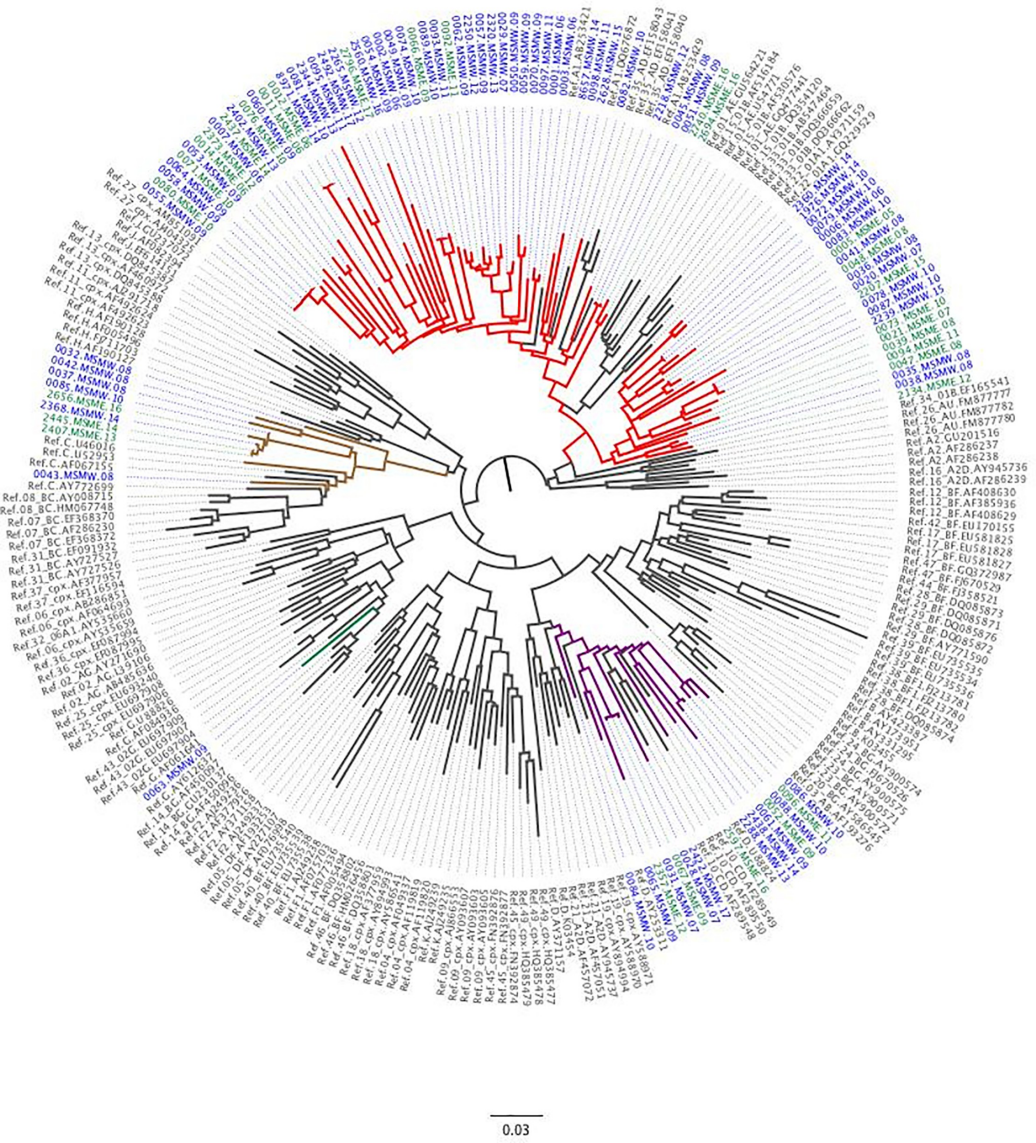
Phylogenetic tree showing relatedness of HIV-1 *pol* sequences from MSM volunteers with acute and early infection from Coastal Kenya, 2005–2017 (N = 97) with reference sequences from the Los Alamos database (N = 158). Tip labels colored according to risk group as follows: grey (references), blue (men who have sex with men exclusively, MSME) and green (men who have sex with men and women, MSMW). Branches are colored according to HIV-1 *pol* subtype inferences as follows: grey (subtype references), red (subtype A1), purple (subtype D), brown (subtype C) and green (subtype G). Table showing a summary of HIV-1 pol subtype inferences by MSME and MSMW.

Of the 97 volunteer sequences, 72 (74%) clustered with HIV-1 sub-subtype A1 references, 15 (16%) with subtype D references and 9 (9%) with subtype C references (Fig 1[A]). One (1%) volunteer sequence clustered with subtype G references. There were no significant differences in the distribution of HIV-1 *pol* subtype inferences between MSMW and MSME sequences (p = 0.915) (Table 2).

**Table 2:** Comparison of HIV-1 *pol* subtype inferences by men who have sex with men exclusively (MSME) and with women (MSMW) from Coastal Kenya, 2005–2017 (N = 97).

HIV-1 *pol* subtype	MSME, n = 30 (%)	MSMW, n = 67 (%)	Total MSM, n = 97 (%)
A1	22 (73)	50 (75)	72 (74)
C	3 (10)	6 (9)	9 (9)
D	5 (17)	10 (15)	15 (15)
G	0 (0)	1 (1)	1 (1)

### Transmission clusters

#### i) Subtype A

**Fig 2:**
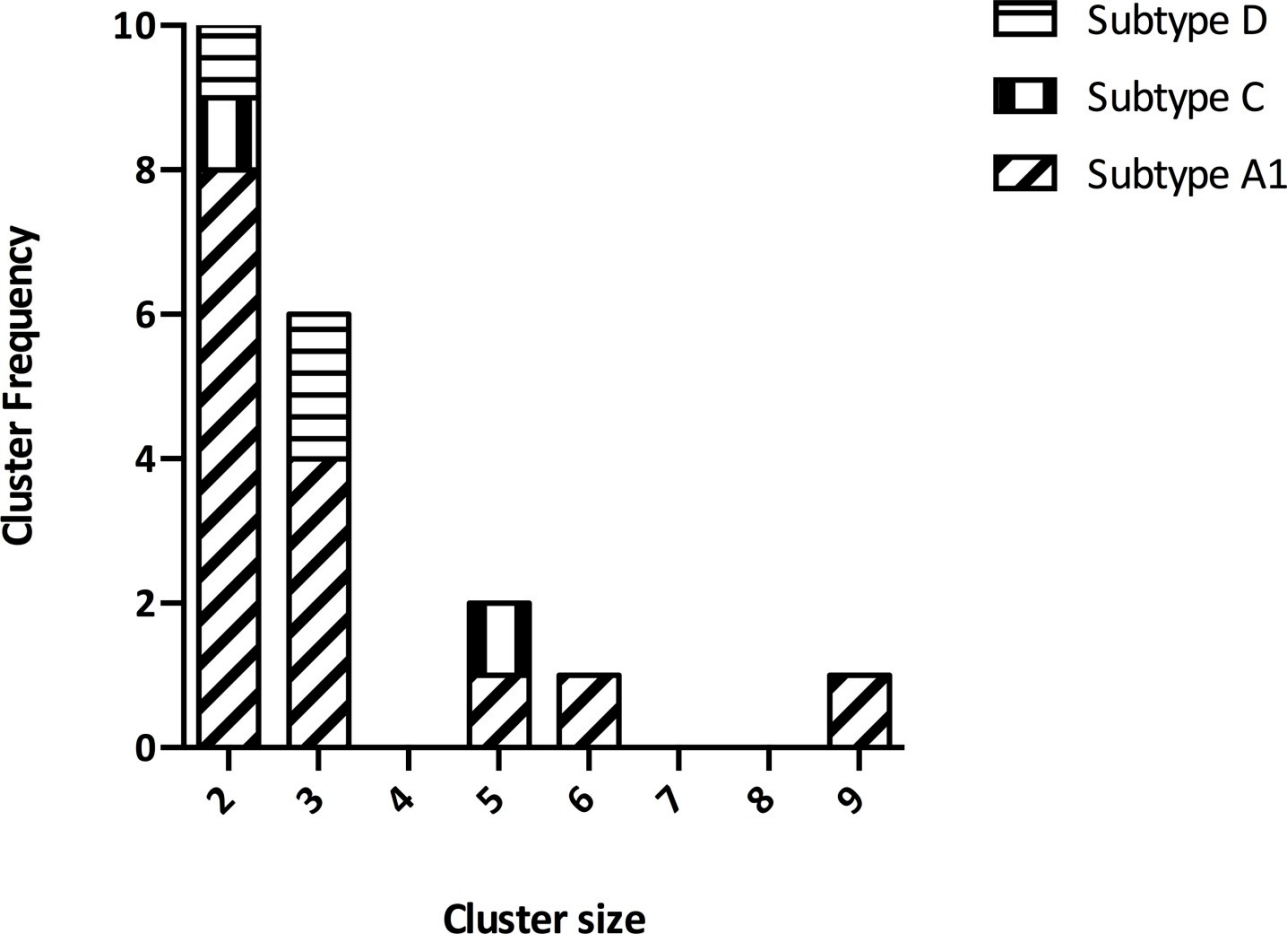
Bar graph showing the distribution of transmission clusters by HIV-1 *pol* subtype amongst acute and recently HIV-1 infected MSM volunteers from Coastal Kenya, 2005–2017 (N = 97).

Of the 72 HIV-1 sub-subtype A1 volunteer sequences, 48 (67%) formed seven networks and eight dyads (Fig 2, S1 Fig). The first cluster network comprised a mix of MSME (n = 1) and MSMW (n = 8) sequences; the first participant was infected in 2007, whilst the last in 2013. The second network comprised a mix of MSME (n = 2) and MSMW (n = 4) sequences; all the six participants were infected within one year of each other, between June 2009 and April 2010. The third network comprised a mix of MSME (n = 2) and MSMW (n = 3) sequences; the first participant was infected in 2007, whilst the last in 2015. The remaining networks were all triads (n = 4); two were MSMW only clusters and two were mixed MSME and MSMW clusters. The eight dyads included MSMW only clusters (n = 5), MSME only clusters (n = 2), and a mixed MSME/MSMW cluster (n = 1).

Of the 72 sub-subtype A1 volunteer sequences, majority were either not related to any of the reference sequences (n = 39 [54%]) or exclusively related with reference sequences of Kenyan (n = 15 [21%]), Ugandan (n = 6 [8%]), Tanzanian (n = 1 [1%]) or US (n = 1 [1%]) origin. The remaining sequences were related to a mix of sequences of either Kenyan/Ugandan/Tanzanian (n = 7 [10%]), Kenya/Tanzania (n = 1 [1%]), Kenya/US (n = 1 [1%]) or US/Finland (n = 1 [1%]) origin (S1 Fig).

Overall, the 72 sub-subtype A1 volunteer sequences had a mean genetic distance of 0.019 nucleotide substitutions/site. There was no significant difference between the mean genetic distance of MSME and MSMW sequences (0.024 vs 0.017 nucleotide substitutions/site, respectively, p = 0.218).

#### ii) Subtype C

Of the nine subtype C volunteer sequences, 7 (78%) formed a network and a dyad (Fig 2, S2 Fig). The network comprised a mix of MSME (n = 3) and MSMW (n = 2) sequences; the first participant was infected in 2010, whilst the last in 2016. The dyad comprised of a MSMW only cluster. Majority of the subtype C sequences were not related to any of the reference sequences (n = 5 [56%]). The remaining sequences were related to reference sequences of either Zimbabwe (n = 2 [22%]), Burundi (n = 1 [11%]) or Philippines (n = 1 [11%]) origin (S2 Fig).

Overall, the nine sub-subtype C volunteer sequences had a mean genetic distance of 0.014 nucleotide substitutions/site. There was no significant difference between the mean genetic distance of MSME and MSMW volunteers (0.003 vs 0.019 nucleotide substitutions/site, respectively, p = 0.211).

#### iii) Subtype D

Of the 15 subtype D volunteer sequences, 8 (53%) formed two networks and one dyad (Fig 2, S3 Fig). Both networks were triads and comprised mixed MSME/MSMW clusters. The dyad also comprised a mixed MSME/MSMW cluster. Majority of the subtype D sequences were not related to any of the reference sequences (n = 10 [67%). The remaining sequences were related to reference sequences of either Kenyan (n = 3 [20%]) or Ugandan (n = 1 [7%]) origin. One sequence (7%) was related to a sequence mix of both Kenyan and Ugandan origin (S3 Fig).

Overall, the 15 sub-subtype D volunteer sequences had a mean genetic distance of 0.021 nucleotide substitutions/site. There was no significant difference between the mean genetic distance of MSME and MSMW volunteers (0.019 vs 0.021 nucleotide substitutions/site, respectively, p = 0.849).

### Transmitted drug resistance

Overall, sequences from eight (8.2% [95% CI: 3.6–15.6]) volunteers had at least one detectable TDR mutation against Nucleoside Reverse Transcriptase Inhibitors (NRTI; n = 2 [2.1%]) and Non-Nucleoside Reverse Transcriptase Inhibitors (NNRTI; n = 7, [7.2%]). One volunteer had dual-class NRTI (M184V) and NNRTI (Y181C) resistance mutations. All the mutations were observed in volunteers infected with HIV-1 sub-subtype A1 (Table 3). None of the volunteers had more than one drug class specific mutation, nor thymidine analogue-associated mutations.

**Table 3:** Distribution of HIV-1 transmitted drug resistance mutations amongst antiretroviral treatment naïve acute and recently HIV-1 infected MSM volunteers from Coastal Kenya, 2005–2017 (N = 97).

Volunteer	Risk group	Age at EDI	Year of EDI	HIV-1 subtype	PI mutations	NRTI mutations	NNRTIs mutations
0014	MSME	26.4	2006	A1	none	D67N	none
0059	MSMW	25.3	2009	A1	none	none	K101E
0078	MSMW	27.1	2010	A1	none	none	K103N
0087	MSMW	26.7	2010	A1	none	none	K103N
0098	MSMW	27.2	2011	A1	none	M184V	Y181C
2239	MSMW	28.7	2014	A1	none	none	K103N
2402	MSMW	24.5	2013	A1	none	none	K103N
2437	MSME	22.6	2014	A1	none	none	K103N

**Abbreviations**: EDI (estimated date of infection), MSME (men who have sex with men, exclusively), MSMW (men who have sex with men, and women), PI (Protease inhibitors), NRTI (Nucleoside Reverse Transcriptase Inhibitors), NNRTI (Non-Nucleoside Reverse Transcriptase Inhibitors).

The most prevalent TDR mutation was the K103N mutation (n = 5). The HIV-1 *pol* sequences from these volunteers formed two highly supported transmission clusters of three and two sequences each (S4 Fig). The first cluster comprised of MSMW only volunteers infected between 2010 and 2015. The second cluster comprised of mixed MSMW/MSME volunteers infected in 2013 and 2014.

There was evidence to suggest that older volunteers had higher TDR levels compared to the younger volunteers (25.0–39.9 vs. 18.0–24.9 years; 15.0% vs 3.5%, p = 0.043). A temporal increase in the prevalence of TDR was also observed, from an estimated 3.9% in 2005–2008 to 13.6% in 2013–2017. However, this did not attain statistical significance (p = 0.470). There were also no statistically significant TDR differences between MSME and MSMW volunteers (6.7% vs 9.0%, p = 0.705) (Table 4).

**Table 4:** Risk factors of HIV-1 transmitted drug resistance amongst acute and early infected MSM volunteers from Coastal Kenya, 2005–2017 (N = 97).

Characteristics	Categories	TDR, n/N (%)	p-value
Risk group	MSM-E MSM-W	2/30 (6.7) 6/67 (9.0)	0.705
Age group at EDI (years)	18.0–24.9 25.0–39.9	2/57 (3.5) 6/40 (15.0)	0.043
Education	Primary, lower Secondary, higher	3/45 (6.7) 5/52 (9.6)	0.599
Year of sample collection	2005/08 2009/12 2013/17	1/26 (3.9) 4/49 (8.2) 3/22 (13.6)	0.470
EDI to sample collection	Less than 1 month More than 1 month	2/51 (3.9) 6/46 (13.0)	0.103
*Recent group sex	No Yes	7/78 (9.0) 1/19 (5.3)	0.598
*Recent transactional sex	No Yes	1/29 (3.5) 7/68 (10.3)	0.262
**Recent alcohol use	No Yes	1/37 (2.7) 7/60 (11.7)	0.119
***Recent total sex partners	≤ 2 partners > 2 partners	5/71 (7.0) 3/26 (11.5)	0.476

**Abbreviations**: EDI (estimated date of infection), HET (heterosexual), MSM-E (men who have sex with men, exclusive), MSM-W (men who have sex with men, and women), MSW-E (men who have sex with women, exclusive), WSM-E (Women who have sex with men, exclusive)

*Group sex/transactional sex in the three months preceding the positive HIV-1 diagnosis.

**Alcohol use in the one month preceding the positive HIV-1 diagnosis.

***Total sex partners: in the one week preceding the positive HIV-1 diagnosis.

## Discussion

While little mixing has been reported between MSM and the general population in Kenya [9], our findings now demonstrate much intermingling between MSME and MSMW in Coastal Kenya. This is evident from the significant mixing of both MSME and MSMW sequences observed in most of the cluster networks, and the consistent comparability in genetic distance between the two groups. In addition, there were no significant differences between HIV-1 subtype distribution and transmitted drug resistance between MSME and MSMW. Our data therefore suggest that the HIV-1 MSME and MSMW epidemics are of a homogenous molecular characterization, and that the differential HIV-1 acquisition risk is likely behavioral, including a higher frequency of receptive anal intercourse among MSME as reported earlier [12].

Overall, our data also confirm that the HIV-1 MSM epidemic from Coastal Kenya is largely characterized by a predominance of sub-subtype A1 infections, which is consistent with subtype diversity literature from Kenya [13–18]. However, and while some studies have also reported high and/or increasing circulating or complex recombinant forms amongst the general heterosexual population [19–22], this was not evident in our study population. The absence of recombinants in this HIV-1 infected MSM population likely complements observations from a previous study reporting little mixing between the general heterosexual and MSM population [9]. However, subtype inferences in our analyses were based on partial HIV-1 *pol* genome data, and the possibility that we may have missed recombination breakpoints occurring outside the *pol* genome cannot be ruled out. Indeed, full genome analysis of 13 MSM isolates from Coastal Kenya reported four unique recombinant forms, with sub-subtype A1 related segments reported in most of their *pol* region, but C and D segments occurring in *vpu*, *gag*, *env* and *nef* [44].

About two-thirds of the volunteer sequences formed phylogenetically linked clusters, with little genetic variability between related sequences. This is consistent with literature on MSM epidemics from sSA [11] and other more developed settings [8, 38]. While some networks comprised volunteers who were all infected within one year of each other, one network included individuals who were infected over a duration of more than nine years. These findings point towards a high proportion of both active and long-sustained transmission networks. However, the high clustering may also reflect the high sampling density of MSM from our setting. Nonetheless, prevention interventions, including pre-exposure prophylaxis (PrEP), particularly targeting MSM at high risk of being in transmission clusters, are therefore warranted. Early identification and linkage to care of HIV-1 infected but unaware MSM individuals may also positively contribute to the control of the MSM epidemic in this setting.

Majority of our HIV-1 MSM sequences were either not related to any other reference sequence, or closely related to sequences of Kenyan origin. Our data, therefore, suggest that the HIV-1 MSM epidemic in Coastal Kenya is likely predominantly of local origin, and not necessarily imported from outside Africa. Not surprisingly, the few remaining volunteer sequences were mostly linked to sequences from other East African countries including the subtype D predominant Uganda, subtype A predominant Tanzania, and subtypes C predominant Burundi and Zimbabwe. This is likely a reflection of the extensive transport and commerce networks by road and railways through the Coastal town of Mombasa, which has been postulated to contribute to the spread of HIV-1 in East Africa [21, 45, 46]. Only four volunteer sequences suggested genetic relatedness with references from outside Africa, including the United States of America, Finland and Philippines. It is possible that these references may be from Kenyans who may have travelled or immigrated to these countries, or that residents from these countries got infected whilst visiting Coastal Kenya, a common tourist destination.

Our data also suggest a possible TDR increase, from low (<5%) to intermediate (5–15%) level resistance, over the last decade amongst HIV-1 infected MSM in Coastal Kenya, which is consistent with other studies from the general population in Kenya [26]. The intermediate level resistance in our early and recently infected MSM population is consistent with estimates from HIV-1 newly diagnosed heterosexual adults [24] but in contrast with low TDR levels reported in volunteers with chronic HIV-1 infection [27], all from the same setting. This may reflect decreased viral fitness of transmitted resistance variants during early infection, and subsequent replacement by wild-type virus with better replicative capacity in the absence of selection from ART in chronic infection [47, 48].

Observed TDR mutations were non-complex, with a predominance of the common NNRTI-based K103N mutation. The high level of K103N mutation is consistent with literature from Kenya [23, 24, 26], and has been attributed to the widespread use of NNRTI in first line ART and the historic use of Nevirapine monotherapy in the prevention of mother to child transmission. NNRTIs have a low genetic barrier [49] and mutations may persist for long durations [50], making them easy to be onward transmitted. Indeed, all our observed K103N mutations formed two phylogenetically related transmission clusters spanning over several years, suggesting that they may have been in circulation for a while, and were unsuspectingly propagated onward in the community as new infections. This observation has also been reported in other developed settings [51, 52]. Focus on interventions towards early identification of infected MSM and linkage to care are therefore warranted, and may contribute to a subsequent reduction in transmitted NNRTI resistance mutations.

The main strength of our study is the use of data and samples from a well-characterized acute and early infected MSM cohort collected over a duration of more than 10 years from a sSA setting. However, our study is not without limitations. Firstly, the small sample size limited our effort for an in-depth analysis to delineate temporal effects and associations by subtypes, transmission clusters and transmitted drug resistance. Secondly, we only included data and samples from MSM populations and were therefore unable to make systematic comparisons of subtype distribution, transmission clusters and TDR between MSM and the general heterosexual population. Lastly, we only used MSM data and samples from one setting along Coastal Kenya, which limits the extent to which our findings may be generalized at the national level or in other regions.

In conclusion, and limitation notwithstanding, our data from a HIV-1 early and acute infected MSM population suggest that this concentrated epidemic is characterized with a predominance of HIV-1 sub-subtype A1, likely of Kenyan origin, with many MSM transmission clusters and having intermediate level TDR. The high proportion of both active and long-sustained transmission networks are likely propagated by HIV-1 infected individuals unaware of their HIV-1 status. Targeted HIV-1 prevention, early identification and care interventions are therefore warranted to break the transmission cycle amongst MSM in Coastal Kenya. A bigger, well designed, and nationally representative study aimed at understanding the molecular epidemiology of HIV-1 infection within and between high-risk groups is also critical to inform targeted interventions towards controlling the HIV-1 epidemic in Kenya.

## Supporting information

S1 Table A summary of HIV-1 reference sequences obtained from the BLAST search by country and inferred subtype, and after removal of duplicate and redundant sequences (N = 330). (DOC)

S1 Fig Phylogenetic tree showing clustering of HIV-1 subtype A pol sequences from MSM volunteers with acute and early HIV infection from Coastal Kenya (N = 72) with reference sequences from the Los Alamos database (N = 221). Branches leading to nodes with aLRT-SH support of >0.85 and >0.90 are colored orange and red respectively. Tip labels are colored according to risk group as follows: grey (references), blue (MSMW) and green (MSME).
(PDF)

S2 Fig Phylogenetic tree showing clustering of HIV-1 subtype C pol sequences from volunteers with acute and early HIV infection from Coastal Kenya (N = 9) with reference sequences from the Los Alamos database (N = 44). Branches leading to nodes with aLRT-SH support of >0.85 and >0.90 are colored orange and red respectively. Tip labels are colored according to risk group as follows: grey (references), blue (MSMW) and green (MSME).
(PDF)

S3 Fig Phylogenetic tree showing clustering of HIV-1 subtype D pol sequences from MSM volunteers with acute and early HIV infection from Coastal Kenya (N = 15) with reference sequences from the Los Alamos database (N = 65). Branches leading to nodes with aLRT-SH support of >0.85 and >0.90 are colored orange and red respectively. Tip labels are colored according to risk group as follows: grey (references), blue (MSMW) and green (MSME).
(PDF)

S4 Fig Phylogenetic tree showing clustering of HIV-1 pol sequences with the K103N mutation from MSM volunteers with acute and early HIV infection from Coastal Kenya (N = 5). Branches leading to nodes with aLRT-SH support of >0.85 and >0.90 are colored orange and red respectively. Tip labels are colored according to risk group as follows: blue (MSMW) and green (MSME).
(PDF)
