# Supplementary material for: HIV-1 subtype diversity, transmission networks and transmitted drug resistance amongst acute and early infected MSM populations from Coastal Kenya
Source: PLoS One. 2018 Dec 18;13(12):e0206177. doi: 10.1371/journal.pone.0206177 (PMC6298690; doi:10.1371/journal.pone.0206177)
Supplement: S1 Table — (DOC) [file pone.0206177.s001.doc]

| **Country** | **Subtype A** | **Subtype C** | **Subtype D** | **Total** |
| --- | --- | --- | --- | --- |
| Albania | 1 | 0 | 0 | 1 |
| Austria | 1 | 0 | 0 | 1 |
| Belgium | 0 | 2 | 0 | 2 |
| Botswana | 0 | 1 | 0 | 1 |
| Brazil | 0 | 4 | 0 | 4 |
| Burundi | 1 | 12 | 0 | 13 |
| Canada | 5 | 1 | 0 | 6 |
| China | 1 | 0 | 0 | 1 |
| Cyprus | 1 | 0 | 0 | 1 |
| Denmark | 3 | 0 | 0 | 3 |
| Ethiopia | 0 | 2 | 0 | 2 |
| Finland | 1 | 0 | 0 | 1 |
| Germany | 0 | 1 | 0 | 1 |
| India | 3 | 0 | 0 | 3 |
| Iran | 1 | 0 | 0 | 1 |
| Italy | 1 | 0 | 0 | 1 |
| Kenya | 73 | 2 | 6 | 83 |
| Malawi | 0 | 1 | 0 | 1 |
| Norway | 1 | 0 | 0 | 1 |
| Philippines | 0 | 1 | 0 | 1 |
| Poland | 1 | 0 | 0 | 1 |
| Rwanda | 6 | 0 | 0 | 6 |
| Senegal | 3 | 2 | 0 | 5 |
| South Africa | 1 | 2 | 0 | 3 |
| Spain | 2 | 0 | 0 | 2 |
| Sudan | 0 | 0 | 1 | 1 |
| Sweden | 3 | 0 | 0 | 3 |
| Switzerland | 6 | 0 | 0 | 6 |
| Tanzania | 20 | 2 | 2 | 24 |
| Uganda | 64 | 2 | 54 | 120 |
| United Kingdom | 7 | 1 | 2 | 10 |
| United States | 13 | 2 | 0 | 15 |
| Zambia | 0 | 6 | 0 | 6 |
| Missing | 2 | 0 | 0 | 2 |
| **Total** | **221** | **44** | **65** | **330** |
