# Supplementary figures and images for: HIV-1 subtype diversity, transmission networks and transmitted drug resistance amongst acute and early infected MSM populations from Coastal Kenya
Source: PLoS One. 2018 Dec 18;13(12):e0206177. doi: 10.1371/journal.pone.0206177 (PMC6298690; doi:10.1371/journal.pone.0206177)

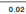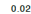

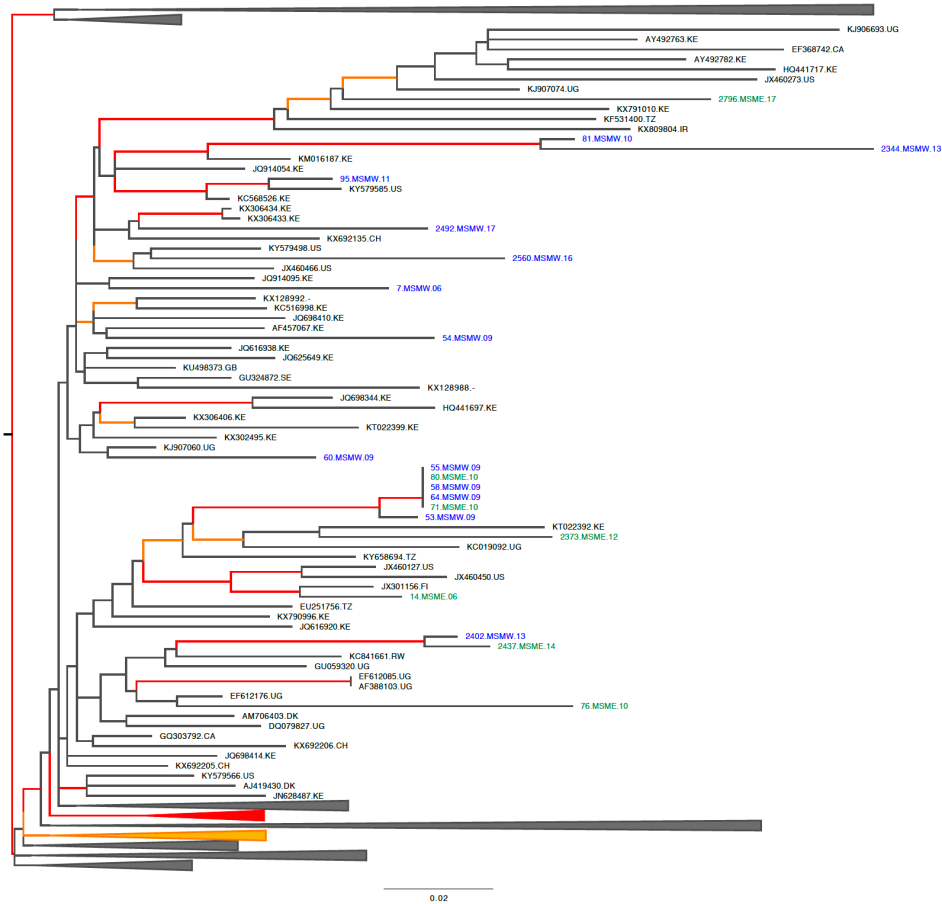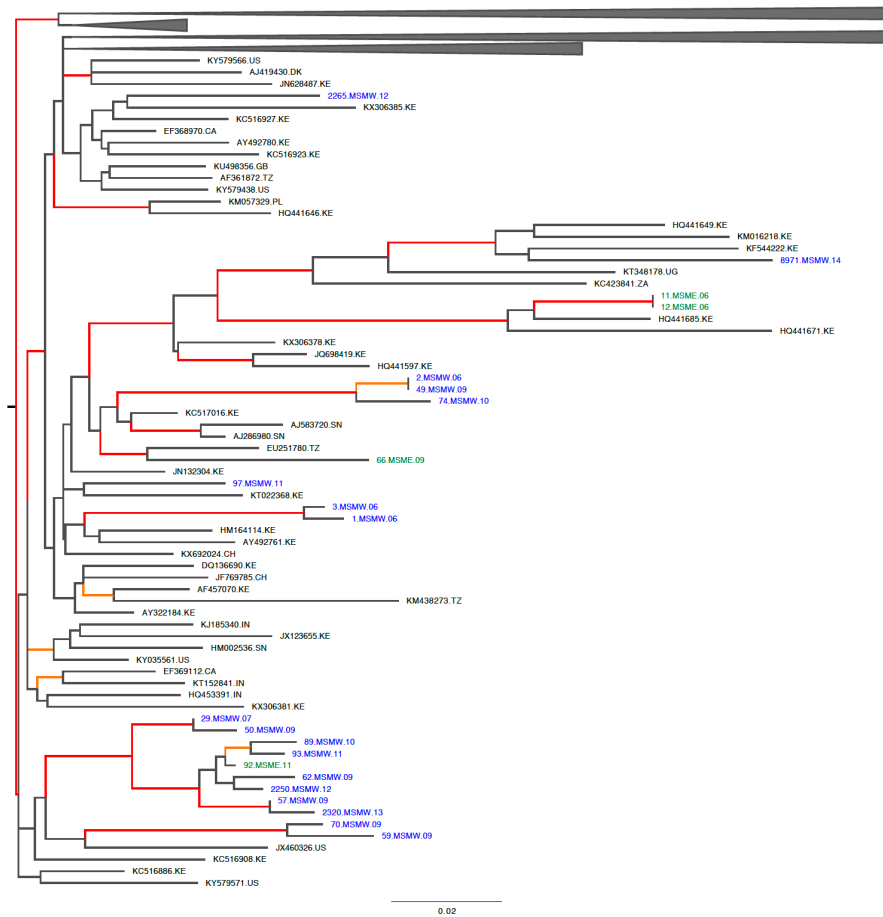

Supplement: S1 Fig — Branches leading to nodes with aLRT-SH support of >0.85 and >0.90 are colored orange and red respectively. Tip labels are colored according to risk group as follows: grey (references), blue (MSMW) and green (MSME). (PDF) [file pone.0206177.s002.pdf]

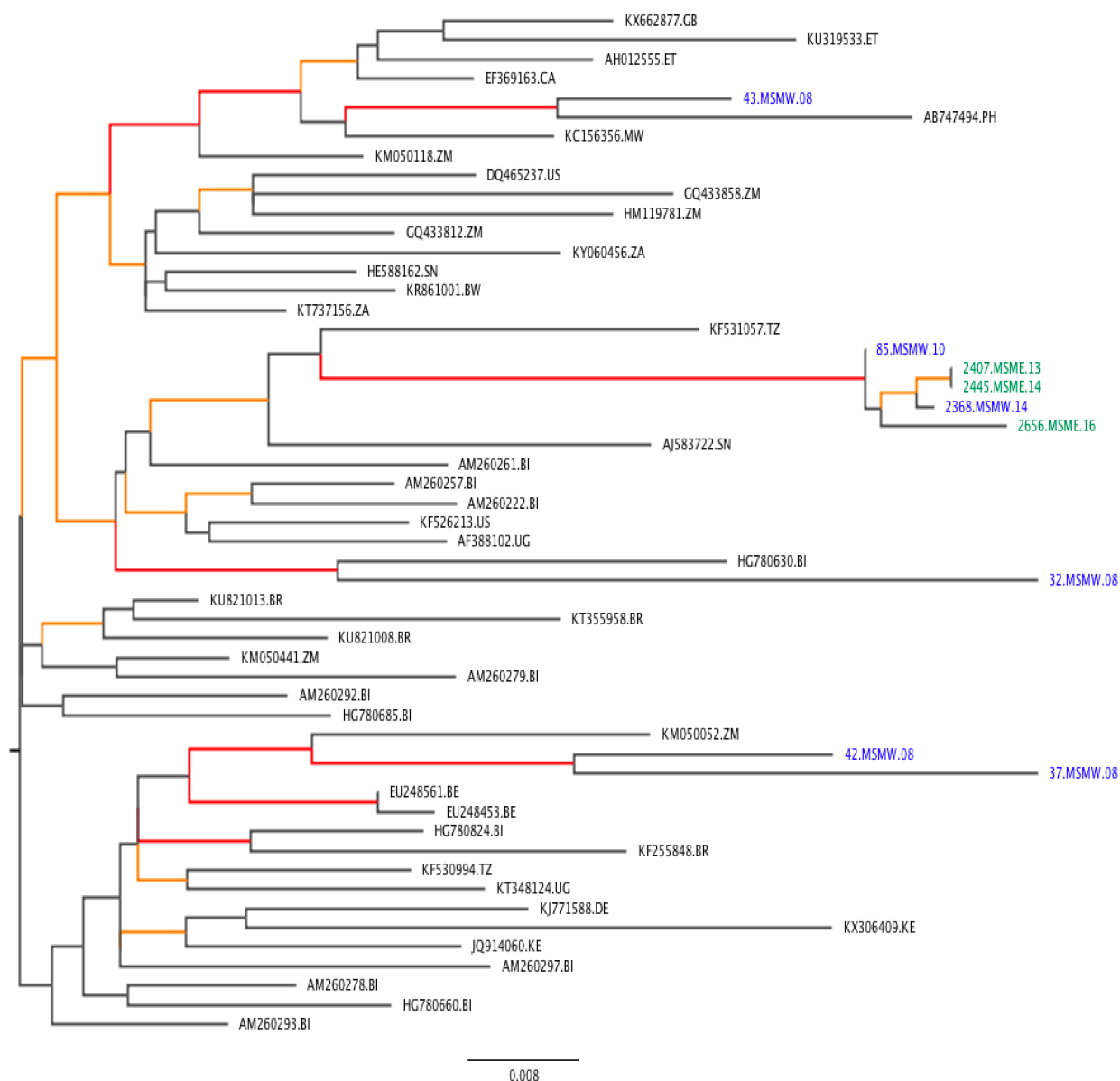

Supplement: S2 Fig — Branches leading to nodes with aLRT-SH support of >0.85 and >0.90 are colored orange and red respectively. Tip labels are colored according to risk group as follows: grey (references), blue (MSMW) and green (MSME). (PDF) [file pone.0206177.s003.pdf]

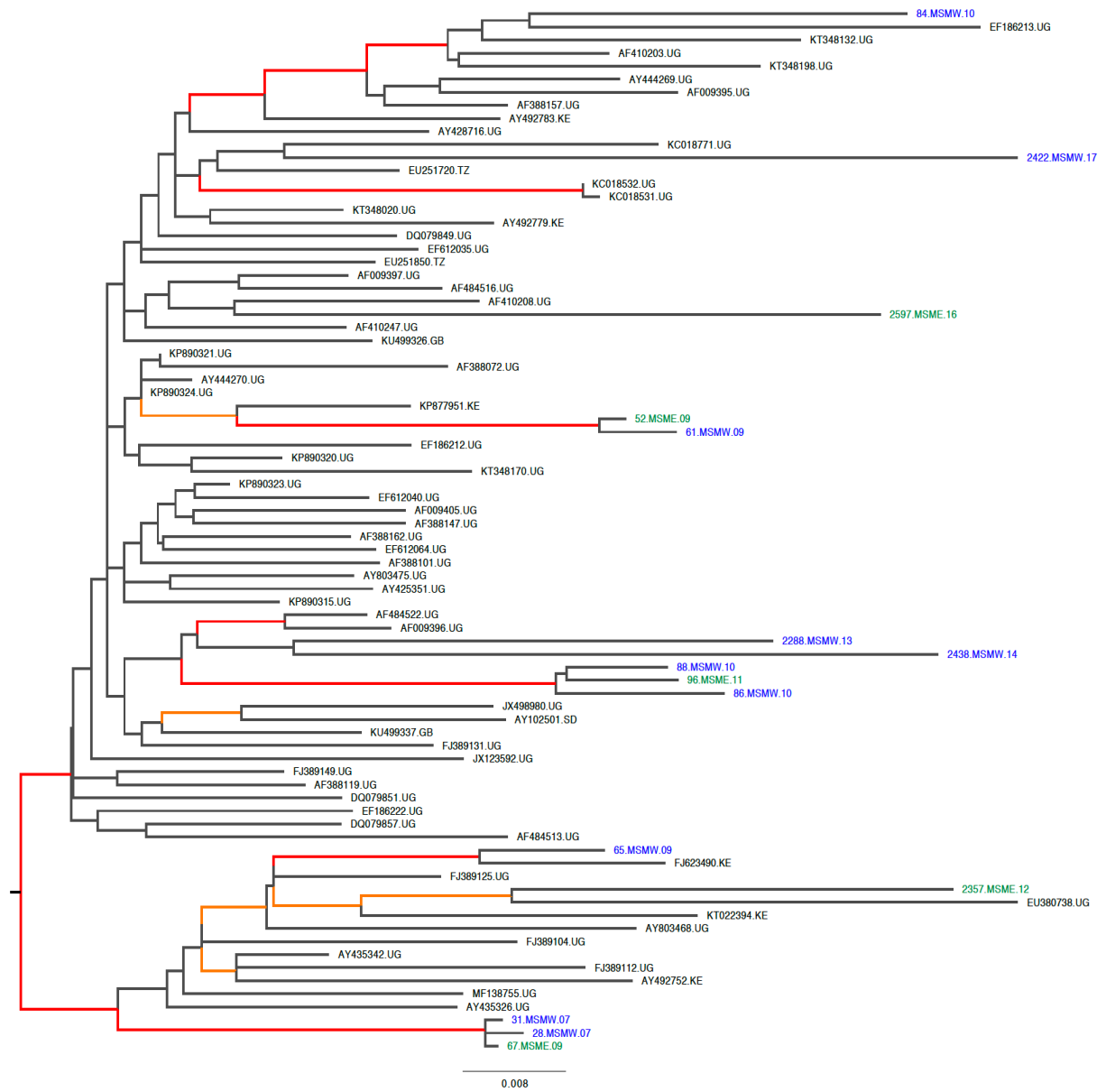

Supplement: S3 Fig — Branches leading to nodes with aLRT-SH support of >0.85 and >0.90 are colored orange and red respectively. Tip labels are colored according to risk group as follows: grey (references), blue (MSMW) and green (MSME). (PDF) [file pone.0206177.s004.pdf]

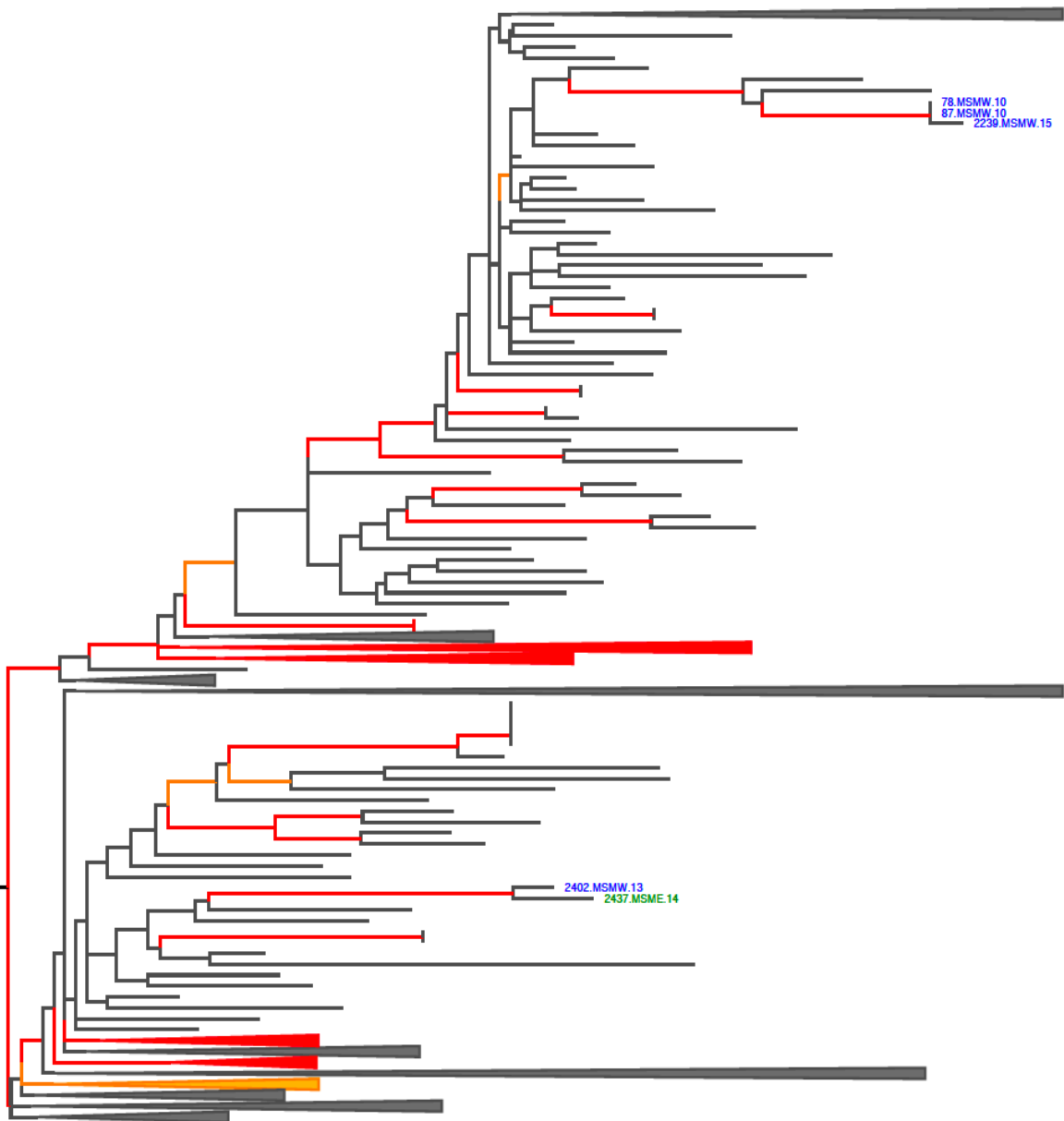

0.02

Supplement: S4 Fig — Branches leading to nodes with aLRT-SH support of >0.85 and >0.90 are colored orange and red respectively. Tip labels are colored according to risk group as follows: blue (MSMW) and green (MSME). (PDF) [file pone.0206177.s005.pdf]
